# Specific targeting of PKCδ suppresses osteoclast differentiation by accelerating proteolysis of membrane-bound macrophage colony-stimulating factor receptor

**DOI:** 10.1038/s41598-019-43501-2

**Published:** 2019-05-07

**Authors:** Mi Yeong Kim, Kyunghee Lee, Hong-In Shin, Daewon Jeong

**Affiliations:** 1 Department of Microbiology, Laboratory of Bone Metabolism and Control, Yeungnam University College of Medicine, Daegu, 42415 Korea; 2 IHBR, Department of Oral Pathology, School of Dentistry, Kyungpook National University, Daegu, 41940 Korea

**Keywords:** Metabolic bone disease, Endocrinology

## Abstract

c-Fms is the macrophage colony-stimulating factor (M-CSF) receptor, and intracellular signalling via the M-CSF/c-Fms axis mediates both innate immunity and bone remodelling. M-CSF-induced transient proteolytic degradation of c-Fms modulates various biological functions, and protein kinase C (PKC) signalling is activated during this proteolytic process via an unknown mechanism. Notably, the role of specific PKC isoforms involved in c-Fms degradation during osteoclast differentiation is not known. Here, we observed that inactivation of PKCδ by the biochemical inhibitor rottlerin, a cell permeable peptide inhibitor, and short hairpin (sh) RNA suppresses osteoclast differentiation triggered by treatment with M-CSF and receptor activator of NF-κB ligand. Interestingly, inhibition of PKCδ by either inhibitor or gene silencing of PKCδ accelerated M-CSF-induced proteolytic degradation of membrane-bound c-Fms via both the lysosomal pathway and regulated intramembrane proteolysis (RIPping), but did not affect c-*fms* expression at the mRNA level. Degradation of c-Fms induced by PKCδ inactivation subsequently inhibited M-CSF-induced osteoclastogenic signals, such as extracellular signal-regulated kinase (ERK), c-JUN N-terminal kinase (JNK), p38, and Akt. Furthermore, mice administered PKCδ inhibitors into the calvaria periosteum exhibited a decrease in both osteoclast formation on the calvarial bone surface and the calvarial bone marrow cavity, which reflects osteoclastic bone resorption activity. These data suggest that M-CSF-induced PKCδ activation maintains membrane-anchored c-Fms and allows the sequential cellular events of osteoclastogenic signalling, osteoclast formation, and osteoclastic bone resorption.

## Introduction

Macrophage colony-stimulating factor (M-CSF) mediates the differentiation of monocytic cells into phagocytic mononuclear macrophages relevant to osteoclast precursors, and subsequently participates in their survival, proliferation, and phagocytic function^1^. The response to M-CSF is mediated by the M-CSF receptor tyrosine kinase, which is encoded by the *c-fms* proto-oncogene^2^. Under normal physiological conditions, the binding of M-CSF to the extracellular domain of c-Fms elicits various signals that are required for the innate immune response, male and female fertility, osteoclast differentiation, and osteoclastic bone resorption^3–5^. In contrast, excessive expression of M-CSF or c-Fms is associated with cancer development and metastasis as well as inflammatory diseases, such as atherosclerosis and rheumatoid arthritis^6–8^. Mice lacking functional M-CSF or c-Fms show an osteopetrotic phenotype due to an osteoclast defect^4,9^. In relation to bone metabolism, the data show that M-CSF and its cognate receptor c-Fms contribute to the proliferation and functional regulation of osteoclast precursor macrophages as well as osteoclast differentiation, and are thereby involved in bone remodelling.

The biological function of the M-CSF/c-Fms axis is primarily regulated by the proteolytic degradation of plasma membrane-anchored c-Fms, which consists of five glycosylated extracellular immunoglobulin (Ig)-like domains, a single transmembrane region, and an intracellular tyrosine kinase domain^10^. When cellular signals induced by various stimulants are transmitted to c-Fms-harboring osteoclast precursor macrophages, c-Fms transiently disappears as a result of proteolytic degradation to restrict signal transduction and the subsequent cellular response^11^. M-CSF, which directly interacts with c-Fms and affects various cellular functions, degrades c-Fms through two distinct lysosomal pathway and regulated intramembrane proteolysis (RIPping). In the lysosomal pathway, the M-CSF/c-Fms complex on the macrophage cell surface undergoes endocytosis and is degraded in the lysosome^12^. Alternatively, c-Fms that becomes dimerised in response to M-CSF is rapidly degraded via RIPping^13^. This process is common for cell surface proteins, such as Fas and Fas ligand, IL-2 and IL-6 receptor, TNFα and receptor activator of NF-κB ligand (RANKL)^14^. In addition, various pro-inflammatory agents, such as non-physiological compound 12-O-tetradecanoylphorbol-13-acetate (TPA; also known as phorbol 12-myristate 13-acetate or PMA)^15^ and pathogen products, such lipopolysaccharide (LPS), lipid A, lipoteichoic acid, and polyI:polyC, that can stimulate Toll-like receptors (TLRs)^16^ can induce RIPping of c-Fms. This is followed by serial cleavage of the extracellular and intracellular domains of c-Fms at the juxtamembrane region by TNF-α-converting enzyme (TACE) and γ-secretase, resulting in ectodomain shedding and release of the intracellular domain into the cytosol. RIPping of c-Fms induced by M-CSF, resulting in ectodomain shedding via TACE, limits the function of M-CSF by reducing receptor availability. After cleavage of the intracellular domain of c-Fms by γ-secretase, it is translocated to the nucleus, where it interacts with transcription factors that induce inflammatory gene expression^17^.

Several intracellular mediators that regulate c-Fms RIPping have been reported. Signalling by phospholipase C and protein kinase C (PKC) is required for the induction of c-Fms RIPping by macrophage activators (*e*.*g*., LPS, IL-2, and IL-4)^18^. In addition, the PKC activator TPA was shown to induce ectodomain shedding of c-Fms and other cell surface proteins, including TNF receptor, IL-6 receptor, CD14, CD16, CD43, and CD44^18,19^. Among the various PKC isoforms, PKCβ and PKCε are involved in the respective regulation of heparin-binding EGF-like growth factor and TNFα shedding^20,21^ and PKCδ and PKCη are involved in regulating IL-6 receptor shedding^22^. These results indicate that PKC signalling may act as a positive regulator of ectodomain shedding during RIPping. In contrast to previous reports, we propose that M-CSF-mediated PKCδ activation negatively regulates lysosomal- and RIPping-dependent proteolytic degradation of the membrane-bound M-CSF receptor c-Fms, thereby retarding c-Fms proteolytic degradation, sustaining M-CSF-induced osteoclastogenic signalling, and stimulating osteoclast differentiation and osteoclastic bone resorption.

## Results

### PKCδ inactivation suppresses osteoclast differentiation

**Figure 1:**
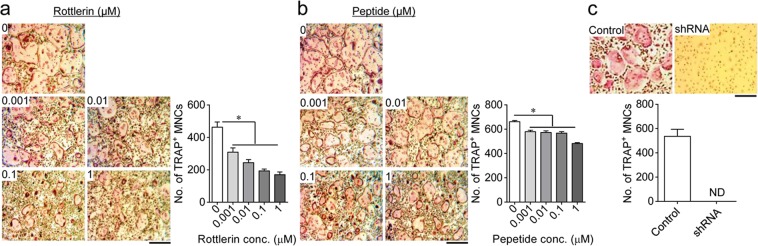
PKCδ inactivation inhibits osteoclast differentiation. (**a**,**b**) Osteoclast precursors were treated with a specific inhibitor of PKCδ (rottlerin or peptide inhibitor; ranging from 0 to 1 μM) or dimethyl sulfoxide (control) and then differentiated into osteoclasts in the presence of M-CSF and RANKL for 4 days. (**c**) Osteoclast precursors were treated with a PKCδ-specific shRNA or a non-targeting shRNA (control) and then differentiated into osteoclasts by culturing with M-CSF and RANKL for 4 days. The extent of osteoclast differentiation was assessed by counting the number of TRAP-positive multinucleated osteoclasts (TRAP^+^ MNCs) with more than three nuclei. Data are mean ± SD (n = 3). *p < 0.01 versus the control. ND, not detected. Scale bar, 500 μm.

We previously reported that M-CSF is critical for osteoclast differentiation, and that it specifically activates PKCα and PKCδ^23^. To determine the role of PKCα and PKCδ signalling in osteoclast differentiation, osteoclast precursors were differentiated into multinucleated osteoclasts in the presence of M-CSF and RANKL for 4 days. Unfortunately, we failed to assess the functional involvement of PKCα in osteoclast differentiation, because the concentration of the specific PKCα inhibitor (Gö6976) that was required to inactivate PKCα had cytotoxic effects during osteoclast differentiation. Therefore, we focused on asking whether M-CSF-mediated PKCδ signalling modulated osteoclast differentiation. Treatment with the cell-permeable biochemical inhibitor rottlerin or the peptide inhibitor that targets PKCδ suppressed osteoclast formation (Fig. 1a,b), with no cytotoxic effects (Supplementary Fig. S1). This finding was also confirmed by PKCδ knockdown using a lentivirus carrying a short hairpin (sh) RNA (Fig. 1c). These results indicated that M-CSF-induced PKCδ activation mediates osteoclast differentiation.

### Blocking of M-CSF-induced PKCδ activation downregulates the M-CSF receptor c-Fms

**Figure 2:**
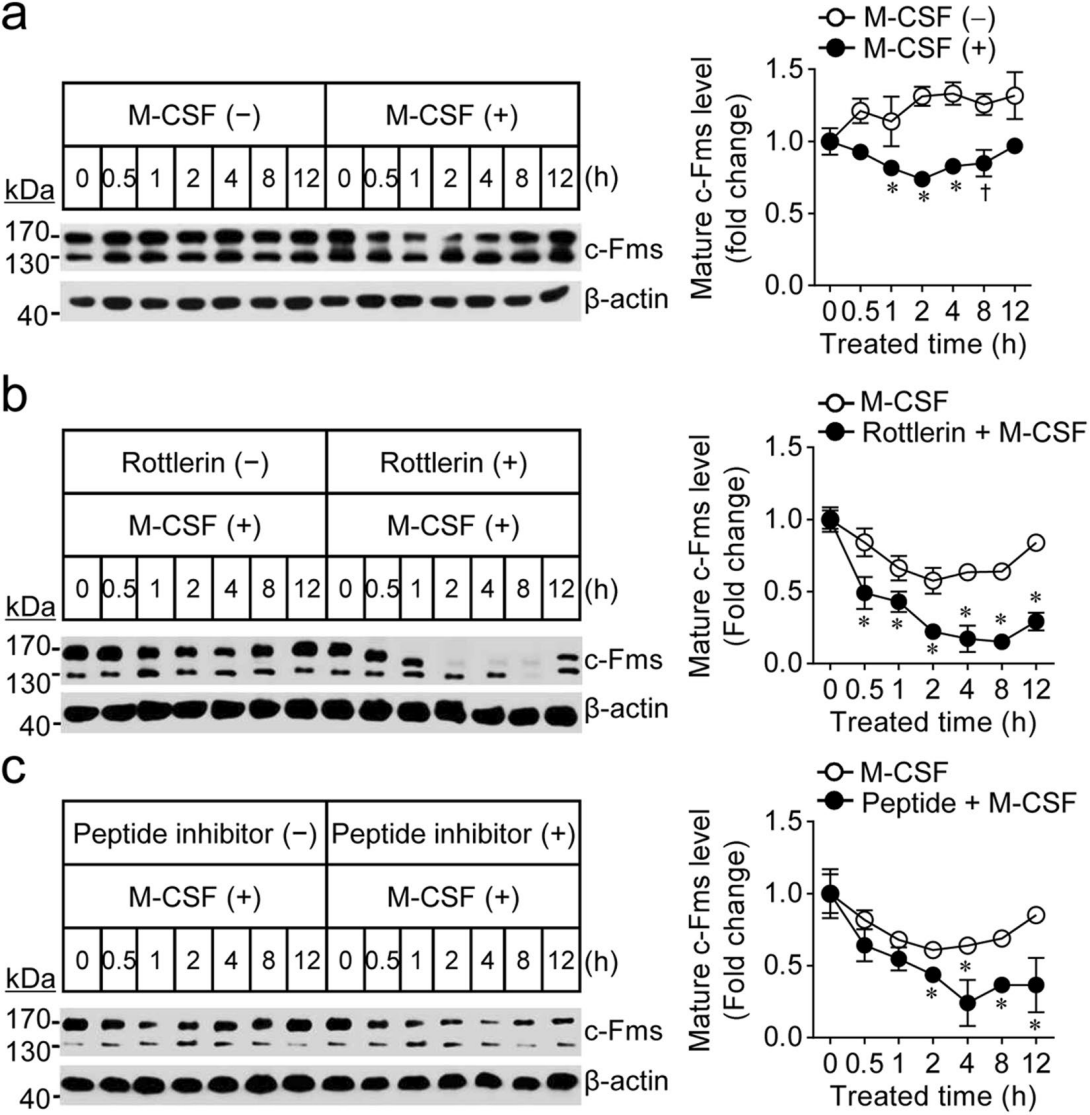
PKCδ inactivation accelerates M-CSF-induced proteolytic degradation of membrane-anchored c-Fms. (**a**–**c**) Osteoclast precursors were incubated in M-CSF-free medium for 4 h and treated with or without a specific inhibitor of PKCδ (rottlerin, 1 μM or peptide inhibitor, 1 nM) for 30 min. Then, the cells were stimulated with or without M-CSF (30 ng/ml) for the indicated times. The levels of c-Fms were evaluated by immunoblot analysis. The fold changes in c-Fms (~170 kDa) were analysed by densitometry and normalised to β-Actin. Data are mean ± SD (n = 3). ^*^p < 0.01, ^†^*p* < 0.05 versus the levels at 0 h.

**Figure 3:**
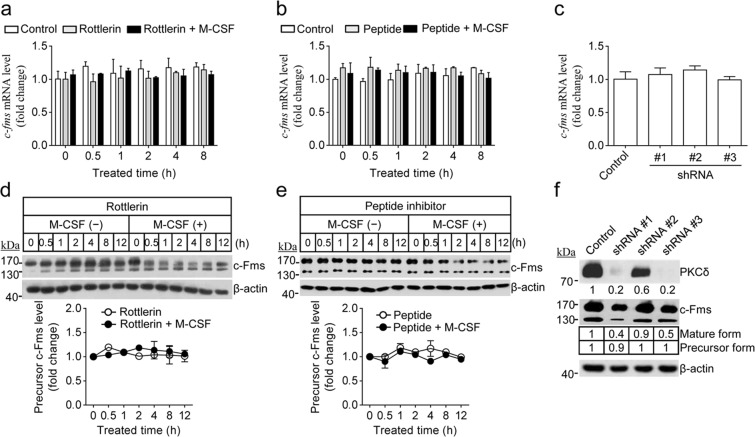
PKCδ inactivation did not affect the mRNA or de novo synthesised protein levels of c-Fms. (**a**–**c**) No change in *c-fms* mRNA levels following PKCδ inactivation. Osteoclast precursors were treated as described in Fig. 2. Then, relative *c-Fms* mRNA levels were analysed by quantitative real-time PCR. Data are mean ± SD (n = 3). (**d**,**e**) After cells were treated as described in Fig. 2a,b, levels of precursor *c-Fms* protein (~130 kDa) were determined by immunoblot analysis. (**f**) Osteoclast precursors treated with three independent PKCδ-specific shRNA clones were incubated with M-CSF for 12 h. Then, the efficiency of PKCδ knockdown and the levels of c-Fms were evaluated by immunoblot analysis. The fold changes in c-Fms (~130 and 170 kDa) and PKCδ were analysed by densitometry and normalised to β-Actin. Data are mean ± SD (n = 3).

Consistent with previous reports^2^, osteoclast precursors have two distinctive M-CSF receptor (c-Fms) types with different molecular weights, a 130 kDa precursor form that is present in the endoplasmic reticulum and a 170 kDa glycosylated mature form that is targeted to the plasma membrane. The mature c-Fms levels slightly decreased in response to M-CSF (Fig. 2a). In osteoclast precursors treated with both PKCδ inhibitor and M-CSF, the levels of mature c-Fms significantly decreased when compared with those treated with M-CSF alone, and then gradually increased to endogenous levels by 12 h after M-CSF exposure (Fig. 2b,c), supporting that PKCδ inactivation accelerates M-CSF-induced c-Fms proteolysis. This decrease was not observed in response to PKCδ inhibitor alone. In addition, we observed that shRNA-mediated PKCδ knockdown led to downregulation of c-Fms, depending on the levels of PKCδ (Fig. 3f). However, PKCδ inactivation did not affect c-Fms precursor protein levels. The combined results indicated that M-CSF-mediated PKCδ signalling maintains the level of mature, membrane-bound c-Fms.

Unexpectedly, we observed that inactivation of PKCδ by rottlerin also led to a progressive decrease in the molecular weight of mature c-Fms (Fig. 2b and Supplementary Fig. S2a,b), whereas inhibition of PKCδ by the peptide blocker or shRNA did not lead to a change in molecular weight. It is known that during maturation, c-Fms undergoes post-translational modifications, most likely *N*-linked glycosylation and phosphorylation^2^. As shown in Supplementary Fig. S2c,d, the reduction in the molecular mass of mature c-Fms induced by rottlerin seems to be dependent on the level of *N*-linked glycosylation, but not phosphorylation.

Next, we tested whether PKCδ inactivation regulates c-Fms at the transcriptional or translational levels. Inactivation of PKCδ by rottlerin, the peptide inhibitor, or shRNA at concentrations that downregulated mature c-Fms protein had no effect on *c-Fms* mRNA levels (Fig. 3a–c). Differing from the transient decrease observed in the mature c-Fms protein after exposure to PKCδ inhibitor or shRNA, c-Fms precursor protein levels did not change (Fig. 3d–f). These results indicated that the reduction in mature c-Fms induced by inhibition of PKCδ may be caused by degradation of membrane-anchored protein.

### Proteolytic degradation of c-Fms induced by PKCδ inactivation leads to a defect in M-CSF-mediated osteoclastogenic signalling

**Figure 4:**
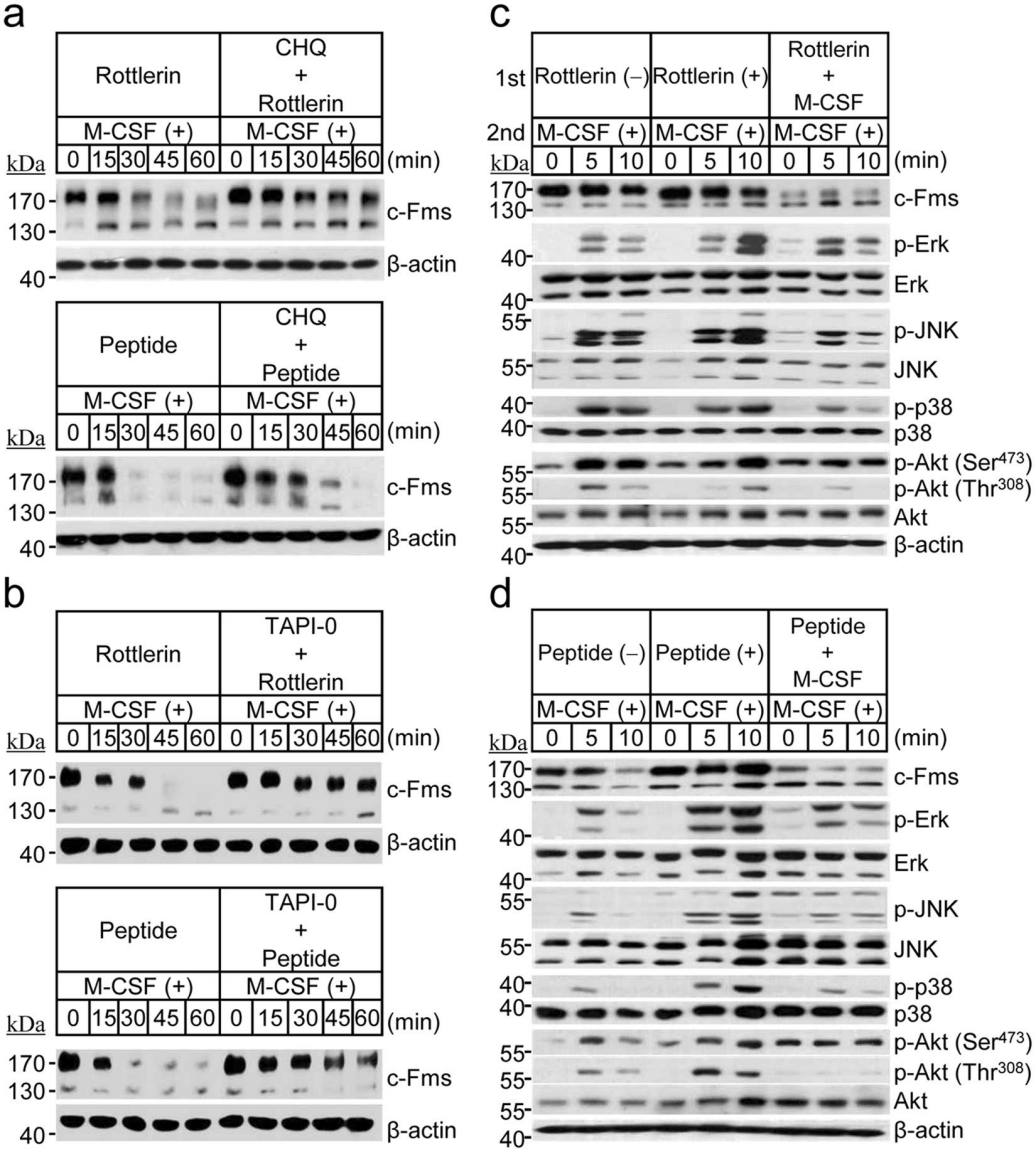
PKCδ inactivation potentiates the proteolytic degradation of c-Fms via both the lysosomal and RIPping pathways and suppresses M-CSF-induced osteoclastogenic signalling. (**a**,**b**) Osteoclast precursors were incubated in the absence of M-CSF for 4 h and then pretreated individually with the indicated inhibitors [PKCδ inhibitors, rottlerin (1 μM) or peptide inhibitor (1 nM); lysosomal inhibitor, chloroquine (CHQ, 20 μM); and an inhibitor of TACE-mediated RIPping, TAPI-0 (100 μM)] for 30 min. Then, the cells were treated with or without M-CSF for the indicated times, and the levels of membrane-anchored c-Fms were determined by immunoblot analysis with an anti-c-Fms antibody. (**c**,**d**) Osteoclast precursors were exposed to M-CSF-depleted condition for 4 h. Firstly, cells were pre-incubated without or with a PKCδ inhibitor (rottlerin or peptide inhibitor) for 30 min and then treated in the presence or absence of M-CSF for 4 h to control the level of c-Fms. Secondly, cells were stimulated with M-CSF for 5 or 10 min and cell lysates were subjected to immunoblot analysis to determine the differential transmission of M-CSF-induced osteoclastogenic signalling. β-Actin was used as a loading control.

Degradation of c-Fms present in the plasma membrane is known occur via lysosomal proteolysis and/or TACE-mediated RIPping^12,13^. Therefore, we analysed the proteolytic process of c-Fms degradation caused by inhibition of PKCδ. The results showed that M-CSF-induced c-Fms degradation induced by inactivation of PKCδ was significantly blocked by treatment with chloroquine, an inhibitor of lysosomal degradation, or TAPI-0, a blocker of TACE-mediated RIPping (Fig. 4a,b), indicating that PKCδ inhibition induces c-Fms degradation via both the lysosomal- and RIPping-dependent pathways. Signalling via the M-CSF/c-Fms axis is known to be associated with osteoclast precursor proliferation and osteoclast survival. Thus, we assessed whether PKCδ inactivation controls M-CSF-mediated osteoclastogenic signalling during proteolytic degradation of c-Fms. As shown in Fig. 4c,d, the strength of the M-CSF-induced signals, including MAPKs and Akt, was markedly suppressed at a time point when c-Fms levels were maximally reduced by exposing both PKCδ inhibitor (rottlerin or peptide inhibitor) and M-CSF. This finding indicated that selective inactivation of PKCδ induced proteolytic degradation of c-Fms and led to a failure of osteoclastogenic signalling transmission via the M-CSF/c-Fms axis.

### PKCδ inactivation inhibits osteoclast formation and bone resorption *in vivo*

**Figure 5:**
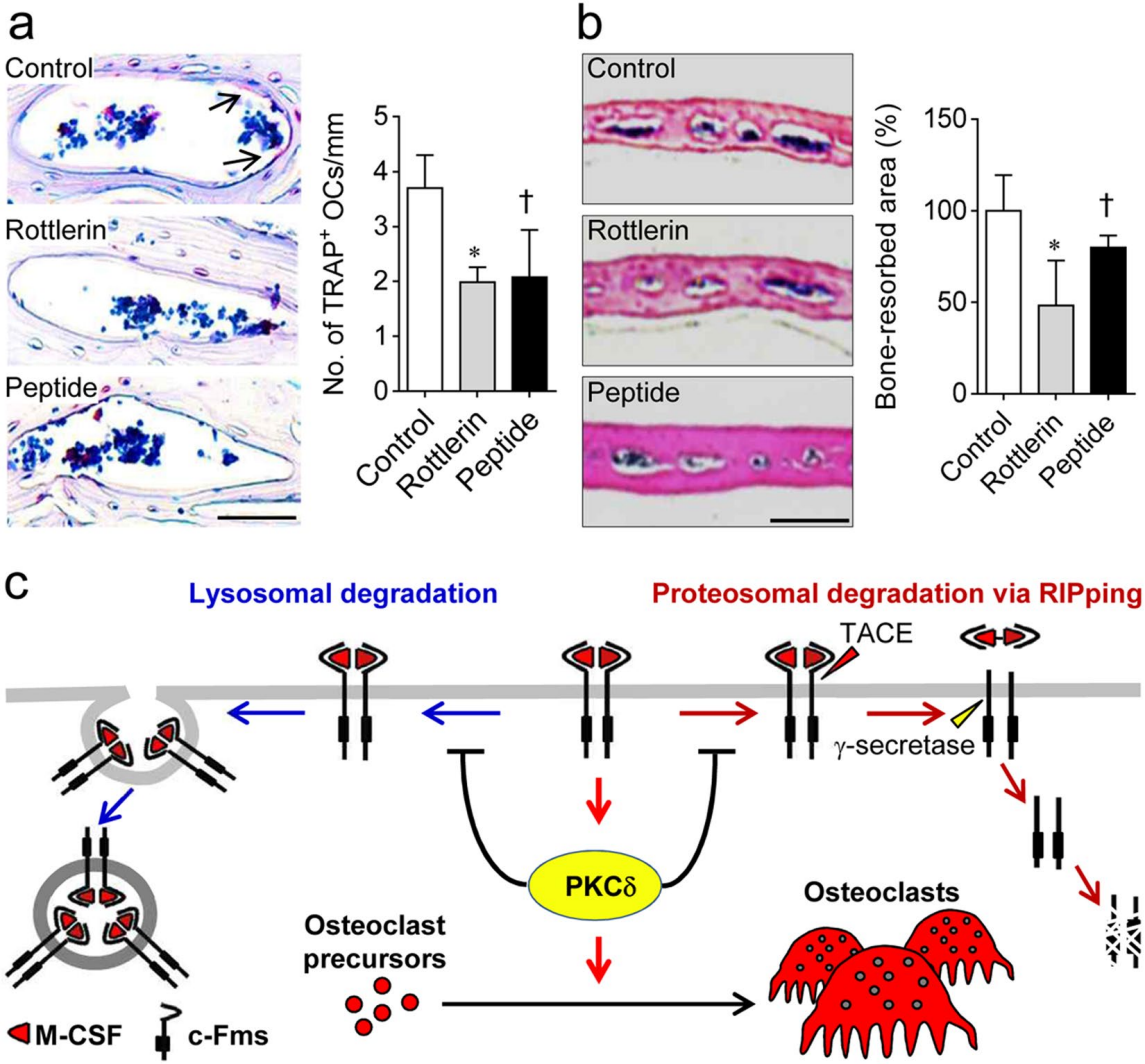
PKCδ inactivation suppresses osteoclast formation and osteoclastic bone resorption *in vivo*. Rottlerin (2 mg/kg), peptide inhibitor (5 mg/kg), or PBS (control) was injected into the calvarial periosteum of the mice daily for 5 days. Three days later, the mice were euthanised, and the calvaria were collected. (**a**) Osteoclast formation in calvaria. The calvarial sections were stained for TRAP, and the number of TRAP-positive osteoclasts on the calvarial bone surface was counted. Arrows indicate TRAP-positive osteoclasts. Scale bar, 50 μm. (**b**) The bone marrow cavity in calvaria. H&E-stained sections were scanned, and the bone marrow cavity was measured. Scale bar, 1 mm. Data are mean ± SD (n = 5). ^*^p < 0.01, ^†^*p* < 0.05 versus the control. (**c**) Schematic representation of the regulatory mechanism underlying the proteolytic degradation of membrane-anchored c-Fms via M-CSF-induced PKCδ activation.

Based on the suppressive effect of PKCδ inhibitor on osteoclast differentiation observed *in vitro*, we analysed whether PKCδ signalling affects osteoclast formation *in vivo*. Mice injected with a PKCδ inhibitor (rottlerin or peptide blocker) into the periosteal region of the calvaria daily for 5 days showed a decrease in the formation of tartrate-resistant acid phosphatase (TRAP)-positive mature osteoclasts on the bone surface within the calvarial bone marrow compared to control mice injected with PBS (Fig. 5a). Further histological analysis of calvarial sections stained with H&E revealed that mice administered the PKCδ inhibitor showed a reduction in the area of the calvarial bone marrow cavity, which reflects the bone-resorbing activity of osteoclasts, compared with this area in control mice (Fig. 5b). These results indicated that a PKCδ inhibitor can suppress osteoclast formation and bone resorption, and thus may be useful as a therapeutic agent for osteoporotic bone loss caused by the excessive bone resorbing activity of osteoclasts.

## Discussion

In this study, we explored the role of PKCδ signalling in M-CSF-induced c-Fms proteolytic degradation during osteoclast differentiation. As shown in Fig. 5c, binding of M-CSF to its cognate receptor c-Fms activates PKCδ, which mediates osteoclast differentiation. M-CSF-mediated PKCδ activation maintains a steady level of membrane-bound c-Fms by preventing its proteolytic degradation via both the lysosomal pathway and RIPping. This sequential process, via the M-CSF/c-Fms/PKCδ axis, sustains M-CSF-induced osteoclastogenic signalling, thereby inducing osteoclast formation and osteoclastic bone resorption under physiological conditions.

We previously showed that M-CSF stimulates PKCδ signalling^23^. Here, we observed that membrane-bound c-Fms is degraded via M-CSF signalling under PKCδ inactivation; however, other forms of c-Fms protein were not degraded following inactivation of PKCδ. These combined results show that M-CSF-induced PKCδ activation controls c-Fms degradation to maintain appropriate levels of membrane-bound c-Fms by mediating intracellular signalling via the M-CSF/c-Fms axis. As shown in Supplementary Fig. S2d, treatment with M-CSF induced higher levels of tyrosine phosphorylation of several proteins, and this phenomenon was verified by adding the phosphatase inhibitor, Na_3_VO_4_, demonstrating that M-CSF led to an increase in kinase activity levels relative to phosphatase activity levels. In addition, PKCδ inhibitor treatment induced a decrease in tyrosine phosphorylation as well as decreased activation of MAPKs and Akt, other serine/threonine-specific protein kinase (Fig. 4c,d; Supplementary Fig. S2d). These findings indicated that M-CSF signalling controls various kinases, including MAPKs, Akt, and PKCδ, and that PKCδ activation participates in the regulation of these downstream kinases. Also, these results indicated that c-Fms regulation via M-CSF-induced PKCδ activation may be modulated by a dynamic interplay between kinases and phosphatases.

Studies have indicated that signalling via the M-CSF/c-Fms axis is tightly associated with the regulation of the immune system, cancer development^24–26^ and bone metabolism. In particular, osteopetrotic (op/op) mice lacking biologically active M-CSF and c-Fms-deficient mice exhibited retarded skeletal growth and osteopetrosis due to osteoclast malfunction^4,9^. Many scientists have attempted to develop therapeutic drugs that target the binding of M-CSF to c-Fms. However, such approaches had limitations, including the lack of a crystal structure for the M-CSF/c-Fms complex^27^. Therefore, alternative therapeutic targets are needed. Here, we showed that selective targeting of PKCδ efficiently blocks osteoclastogenic signalling by accelerating c-Fms degradation, resulting in decreased osteoclast formation. Unexpectedly, we observed that inactivation of PKCδ by rottlerin led to lower molecular weight forms of membrane-bound c-Fms (Supplementary Fig. S2a). This seems to be caused by de-glycosylation of c-Fms, resulting from increased glycosidase activity (Supplementary Fig. S2c). In addition, our data and data from other studies have showed that rottlerin, at higher than nanomolar concentrations which are capable of inhibiting PKCδ, suppresses the activity of other protein kinases^28^. The suppressive effect of rottlerin on protein kinase activity is poorly understood; therefore, it is not known whether PKCδ inactivation by rottlerin downregulates numerous downstream protein kinases or rottlerin acts non-specifically on other protein kinases in addition to PKCδ. For this reason, whether rottlerin is a specific inhibitor of PKCδ is still a matter of debate. However, we also showed that a specific cell-permeable peptide inhibitor of PKCδ did not affect the de-glycosylation of c-Fms, and the fact that a PKCδ-specific peptide inhibitor directly modulates other protein kinases has not been previously reported. Thus, this PKCδ inhibitory peptide may be better than rottlerin for the treatment of patients with osteoporotic bone defects caused by excessive osteoclast formation.

Our data showed that selective inhibition of PKCδ signalling accelerates the proteolytic degradation of the membrane-bound M-CSF receptor c-Fms, and thus suppresses osteoclast differentiation and bone resorption. Thus, exploring anti-osteoclastogenic agents capable of inducing c-Fms degradation will be useful for the development of alternative anti-osteoporotic drugs that can substitute for direct antagonists of c-Fms and inhibitors of the interaction between M-CSF and c-Fms.

## Materials and Methods

### Materials

A biochemical inhibitor (Rottlerin) and a peptide inhibitor [delta PKC (8–17)] that specifically target PKCδ were purchased from Calbiochem (San Diego, CA, USA) and Anaspec, Inc. (Fremont, CA, USA), respectively. Primary antibodies were obtained from the following sources: c-Fms/CSF-1R (C-20), Akt, p-Akt^Ser473^, p-Tyr (PY350), and β-actin from Santa Cruz Biotechnology (Dallas, TX, USA); PKCδ from BD Bioscience (San Jose, CA, USA); and Erk, p-Erk, JNK, p-JNK, p38, p-p38, and p-Akt^Thr308^ from Cell Signalling Technology (Danvers, MA, USA). All common reagents were from Sigma-Aldrich (St. Louis, MO, USA) unless otherwise specified.

#### Preparation of osteoclast precursors and osteoclast differentiation

Osteoclast precursors were isolated from the tibia and femur of 6-week-old C57BL/6 male mice (Koatech, Inc., Gyeonggido, Korea) by flushing the bone marrow as previously described^29^. In brief, erythrocytes within the bone marrow fraction were lysed with red blood cell lysis buffer (Sigma-Aldrich). The remaining cells were cultured in alpha minimum essential medium (α-MEM; Hyclone, Logan, UT, USA) containing 10% foetal bovine serum (FBS, Hyclone), 1% antibiotic-antimycotic solution (Thermo Fisher Scientific, Inc., Waltham, MA, USA), and recombinant human M-CSF (5 ng/ml) for 12 h at 37 °C in 5% CO_2_. To generate osteoclast precursors, the floating cells were further cultured in α-MEM containing M-CSF (30 ng/ml) for 3 days. For osteoclast differentiation, osteoclast precursors (2.5 × 10^4^ cells per well) were seeded onto 48-well culture plates and then differentiated into osteoclasts in the presence of M-CSF (30 ng/ml) and recombinant mouse RANKL (100 ng/ml) for 4 days. The medium was exchanged on day 2. To evaluate osteoclast differentiation, cells were stained for tartrate-resistant acid phosphatase (TRAP) using the Leukocyte Acid Phosphatase Staining Kit (Sigma-Aldrich) according to the manufacturer’s instructions. TRAP-positive multinucleated cells (TRAP^+^ MNCs) with more than three nuclei were counted under a light microscope.

#### Knockdown of PKCδ by short hairpin RNA (shRNA)

Lentiviral pLKO.1-puro expression plasmids encoding shRNA specific for PKCδ were purchased from Sigma-Aldrich (MISSION^®^ shRNA plasmid DNA; clone IDs: NM_011103.1, NM_011103.2, and NM_011103.2). A pLKO.1-puro non-targeting shRNA plasmid was used as a control. Lentiviral particles were generated in HEK293T cells by co-transfection with compatible packaging plasmids encoding VSV-G and NL-BH, and shRNA-encoding plasmids using polyethylenimine (Polysciences, Inc., Warrington, PA, USA). After the culture medium containing lentivirus was passed through a 0.45 μm syringe filter, osteoclast precursors were infected with lentivirus in the presence of polybrene (8 μg/ml; Sigma-Aldrich) for 6 h, and then incubated in fresh medium containing M-CSF (60 ng/ml) and puromycin for selection (1 μg/ml; Sigma-Aldrich) for 2 days. Puromycin-resistant cells were differentiated into osteoclasts in the presence of M-CSF (30 ng/ml) and RANKL (100 ng/ml) for 4 days.

#### Immunoblot analysis

Cells were lysed with a lysis buffer containing 50 mM Tris-HCl, pH 7.4, 150 mM NaCl, 1 mM EDTA, 1% Nonidet-P40, 1% SDS, 1 mM NaF, 1 mM Na_3_VO_4_, 1 mM β-glycerophosphate, and 1× protease inhibitor cocktail (Roche, Mannheim, Germany), and then the cell lysates were centrifuged at 10,000 × *g* for 10 min at 4 °C. The protein concentration of the resulting supernatants was determined using the DC protein assay (Bio-Rad, Hercules, CA, USA). Proteins (20 μg) were separated by 10% SDS-PAGE, transferred to nitrocellulose membranes (GE Healthcare, Pittsburgh, PA, USA), and incubated with the primary antibodies as indicated. The antigen-antibody complexes were detected with appropriate horseradish peroxidase-conjugated secondary antibodies and ECL reagents (Abfrontier, Seoul, Korea). Fold changes were measured using Image J software 1.49 v (NIH, Bethesda, MD, USA).

#### Quantitative Real-time PCR

Total RNA was extracted from the cells using TRIzol reagent (Invitrogen, Carlsbad, CA, USA) and then reverse transcribed into cDNA using the M-MLV Reverse Transcription Kit (Invitrogen). Real-time polymerase chain reaction (PCR) was conducted in triplicate using Lightcycler^®^ 480 SYBR Green I Master Mix (Roche) and a Lightcycler 96 system (Roche). The levels of *c-fms* mRNA were analysed using the comparative delta threshold cycle method using glyceraldehyde-3-phosphate dehydrogenase (*GAPDH*) mRNA as an internal control. PCR primers were synthesised by Bionics Corp. (Seoul, Korea), and their sequences were as follows: mouse *c-fms* forward 5′-CAG AGC CCC CAC AGA TAA AA-3′ and reverse 5′-GTC CAC AGC GTT GAG ACT GA-3′; mouse *GAPDH* forward 5′-AGG TCG GTG TGA ACG GAT TTG-3′ and reverse 5′-TGT AGA CCA TGT AGT TGA GGT CA-3′.

#### Animal study

To assess the effects of the PKCδ inhibitors on calvarial bone remodelling, 8-week-old C57BL/6 male mice purchased from Koatech, Inc. were maintained in the mouse facility. All procedures using the mice were conducted in accordance with the Guide for the Care and Use of Laboratory Animals of Yeungnam University College of Medicine and were approved by the Institutional Animal Research Review Committee of Yeungnam University College of Medicine (permission number YUMC-AEC2016-038). Rottlerin (2 mg/kg), delta PKC (8–17) (5 mg/kg), or PBS (control) was injected onto the calvarial periosteum of the mice every day for 5 days, and then the mice were sacrificed on day 3 after the final injection. Calvarial specimens were surgically dissected from the mice, fixed in 3.7% formaldehyde, decalcified with EDTA solution, and sectioned using a microtome. The sections were then stained with TRAP to detect the osteoclasts on the calvarial bone surface and with haematoxylin and eosin (H&E) to visualise the bone marrow cavity, to assess osteoclastic bone-resorbing activity. The number of TPAP-positive osteoclasts was counted under a light microscope. To measure the area of the bone marrow cavity, which reflects osteoclastic bone-resorbing activity, images were scanned using an Aperio ScanScope (Model T3) and analysed using ImageScope software, version 6 (Aperio Technologies, Vista, CA, USA).

#### Statistical analysis

Quantitative data were expressed as the mean ± SD of at least three independent experiments. Statistical analyses were performed using Student’s two-tailed t-test for comparison between two groups or analysis of variance (ANOVA) and a post-hoc test for multiple comparisons using SPSS 21.0 software. *P* values less than 0.05 were considered statistically significant.

## Supplementary information

Supplemental information

## Data Availability

All data generated or analysed during this study are included in this published article (and its Supplementary Information files).
