# Supplementary material for: Specific targeting of PKCδ suppresses osteoclast differentiation by accelerating proteolysis of membrane-bound macrophage colony-stimulating factor receptor
Source: Sci Rep. 2019 May 7;9:7044. doi: 10.1038/s41598-019-43501-2 (PMC6504882; doi:10.1038/s41598-019-43501-2)
Supplement: Supplementary file 1 — Supplemental information [file 41598_2019_43501_MOESM1_ESM.docx]

**Specific targeting of PKCδ suppresses osteoclast differentiation by accelerating proteolysis of membrane-bound macrophage colony-stimulating factor receptor**

Mi Yeong Kim^1^, Kyunghee Lee^1^, Hong-In Shin^2^ & Daewon Jeong^1^

^1^Department of Microbiology, Laboratory of Bone Metabolism and Control, Yeungnam University College of Medicine, Daegu 42415, Korea

^2^IHBR, Department of Oral Pathology, School of Dentistry, Kyungpook National University, Daegu 41940, Korea

Correspondence: Daewon Jeong, Department of Microbiology, Yeungnam University College of Medicine, 170 Hyunchung-Ro, Nam-Gu, Daegu 42415, Korea. E-mail: [dwjeong@ynu.ac.kr](mailto:dwjeong@sis.snu.ac.kr); Tel.: +82-53-640-6944; Fax: +82-53-657-6869

**Supplementary Figure Legends**

**
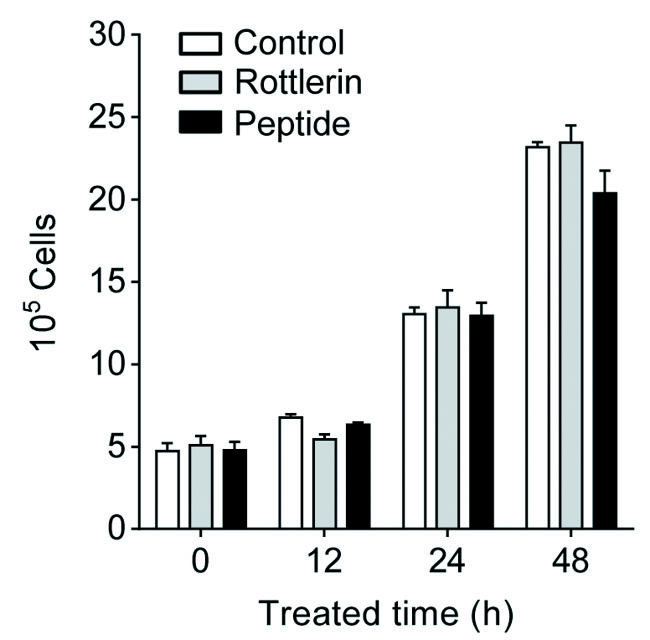
**

**Supplementary Figure 1** **Osteoclast precursor proliferation.** Osteoclast precursors (1 × 10^5^ cells per well in a 12-well culture plate) were treated with rottlerin (1 μM), peptide inhibitor (1 nM), or dimethyl sulfoxide (control) in the presence of M-CSF (30 ng/ml) for the indicated times. After trypsinisation, cells were counted by using the trypan blue dye exclusion method. Data are mean ± SD of a triplicate set.


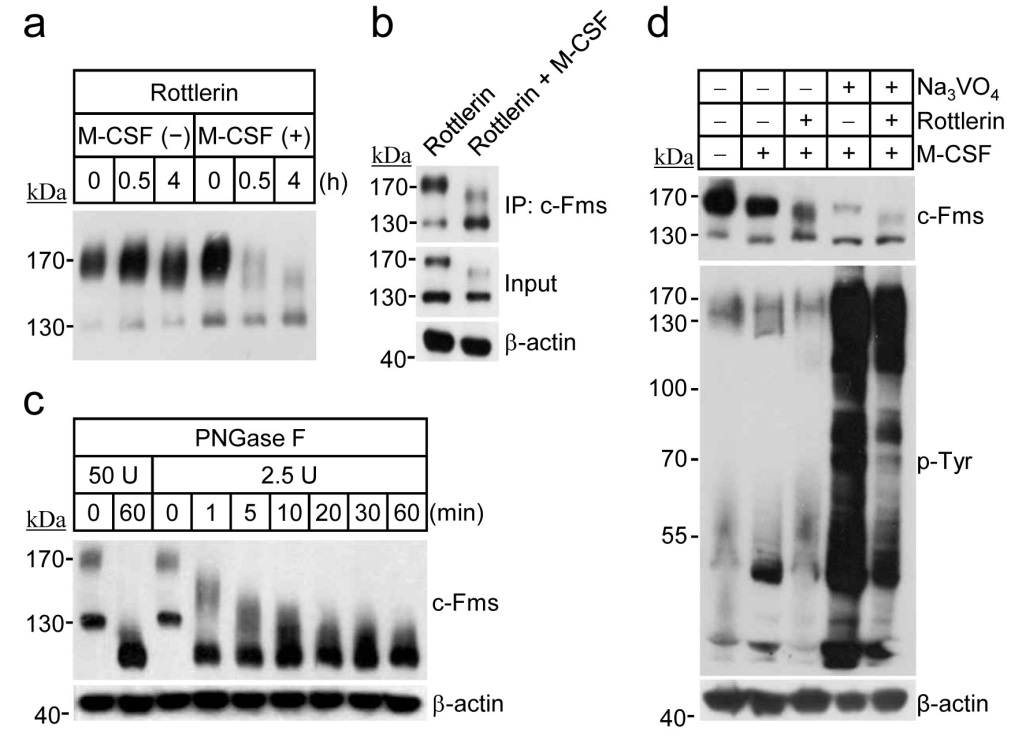


**Supplementary Figure 2** **PKCδ inactivation by rottlerin induces a reduction in the molecular weight of c-Fms due to de-glycosylation, but not de-phosphorylation.** (a) Reduced molecular mass of mature c-Fms. Osteoclast precursors were incubated without M-CSF for 4 h, treated with rottlerin (1 μM) for 30 min, and then stimulated with or without M-CSF (30 ng/ml) for the indicated times. Cell lysates were electrophoresed on a 10% SDS-PAGE to separate the high molecular mass proteins and then subjected to immunoblot analysis with a specific antibody against c-Fms. (b) Immunoprecipitation of c-Fms. Osteoclast precursors were treated with rottlerin (1 μM) in the absence or presence of M-CSF for 4 h and lysed with a lysis buffer (20 mM Tris-HCl, pH 7.5, 150 mM NaCl, 1% NP40, and 1× protease inhibitor). Then, the cell lysates were immunoprecipitated with an anti-c-Fms antibody and protein A/G agarose and subjected to immunoblot analysis with an anti-c-Fms antibody. (c) c-Fms de-glycosylation. Cell lysates were prepared from osteoclast precursors by using recombinant PNGase F kit according to the manufacturer’s protocol (New England BioLabs, Inc., Ipswich, MA, USA) and then reacted with peptide N-glycosidase F (PNGase F) for the indicated times. The reacted samples were resolved on a 10% SDS-PAGE, and the change in the molecular mass of de-glycosylated c-Fms was determined by immunoblot analysis. (d) c-Fms without or with phosphorylation. Osteoclast precursors were pretreated with rottlerin (1 μΜ) and/or a phosphatase inhibitor (Na_3_VO_4_, 1 mM) for 30 min and then stimulated with M-CSF (30 ng/ml) for 4 h. The difference in the molecular mass of c-Fms and the relative amount of tyrosine (Tyr) phosphorylated proteins were analysed by immunoblot analysis with anti-c-Fms and anti-phospho-Tyr (p-Tyr) antibodies, respectively.
